# Multicenter Clinical Performance Evaluation of Omadacycline Susceptibility Testing of *Enterobacterales* on VITEK 2 Systems

**DOI:** 10.1128/jcm.00174-23

**Published:** 2023-05-10

**Authors:** Edith Csiki-Fejer, Maria Traczewski, Gary W. Procop, Thomas E. Davis, Meredith Hackel, Hari P. Dwivedi, David H. Pincus

**Affiliations:** a bioMérieux, Inc., Hazelwood, Missouri, USA; b Clinical Microbiology Institute, Inc., Wilsonville, Oregon, USA; c Cleveland Clinic, Cleveland, Ohio, USA; d Indiana University School of Medicine Indianapolis, Indiana, USA; e IHMA Inc., Schaumburg, Illinois, USA; NorthShore University HealthSystem

**Keywords:** Antimicrobial Susceptibility Testing, VITEK 2 AST-GN omadacycline, *Enterobacterales*

## Abstract

We present the first performance evaluation results for omadacycline on the VITEK 2 and VITEK 2 Compact Systems (bioMérieux, Inc.). The trial was conducted at four external sites and one internal site. All sites were in the United States, geographically dispersed as follows: Indianapolis, IN; Schaumburg, IL; Wilsonville, OR; Cleveland, OH; and Hazelwood, MO. In this multisite study, omadacycline was tested against 858 *Enterobacterales* on the VITEK 2 antimicrobial susceptibility test (AST) Gram-negative (GN) card, and the results were compared to the Clinical and Laboratory Standards Institute broth microdilution (BMD) reference method. The results were analyzed and are presented as essential agreement (EA), category agreement (CA), minor error (mE) rates, major error (ME) rates, and very major error (VME) rates following the US Food and Drug Administration (FDA) and International Standards Organization (ISO) performance criteria requirements. Omadacycline has susceptibility testing interpretive criteria (breakpoints) established by the FDA only; nevertheless, the analysis was also performed using the ISO acceptance criteria to satisfy the registration needs of countries outside the United States. The analysis following FDA criteria (including only Klebsiella pneumoniae and Enterobacter cloacae) showed the following performance: EA = 97.9% (410/419), CA = 94.3% (395/419), VME = 2% (1/51), with no ME present. The performance following ISO criteria (including all *Enterobacterales* tested) after error resolutions was EA = 98.1% (842/858) and CA = 96.9% (831/858). No ME or VME were observed. The VITEK 2 test met the ISO and FDA criteria of ≥ 95% reproducibility, and ≥ 95% quality control (QC) results within acceptable ranges for QC organisms. In June 2022, the omadacycline VITEK 2 test received FDA 510(k) clearance (K213931) FDA as a diagnostic device to be used in the treatment of acute bacterial skin and skin-structure infections caused by E. cloacae and K. pneumoniae, and for treatment of community-acquired bacterial pneumonia caused by K. pneumoniae. The new VITEK 2 AST-GN omadacycline test provides an alternative to the BMD reference method testing and increases the range of automated diagnostic tools available for determining omadacycline MICs in *Enterobacterales*.

## INTRODUCTION

Omadacycline, commercialized under the brand name Nuzyra is a broad-spectrum oral and intravenous antibiotic approved by FDA in 2018 for the treatment of adult patients suffering from acute bacterial skin and skin-structure infections (ABSSSI) and community-acquired bacterial pneumonia (CABP) infections (1) based on results of 3, phase 3, double-blind, randomized clinical trials, 2 for ABSSSI and 1 for CABP (2–4).

Omadacycline effectively treats ABSSSI caused by Staphylococcus aureus (methicillin-susceptible and resistant isolates), Staphylococcus lugdunensis, Streptococcus pyogenes, Streptococcus anginosus group (includes *S. anginosus*, S. intermedius, and *S. constellatus*), Enterococcus faecalis, Enterobacter cloacae, and Klebsiella pneumoniae (1).

Also, omadacycline effectively treats CABP caused by Streptococcus pneumoniae, S. aureus (methicillin-susceptible isolates), Haemophilus influenzae, Haemophilus parainfluenzae, and K. pneumoniae (1). Moreover, omadacycline is active against atypical organisms causing CABP Legionella pneumophila, Mycoplasma pneumoniae, and Chlamydophila pneumoniae, conferring an advantage over beta-lactams, not helpful in those circumstances (1, 5).

Omadacycline is part of the third-generation tetracycline antibiotic class, along with tigecycline and eravacycline. The structure of the aminomethylcycline omadacycline consists of the napththacene tetracycline core, 6-deoxy-6-demethyltetracycline, which is essential for antimicrobial activity (6), and modifications (compared with tetracycline) at the C7 position (dimethylamino group - same as in minocycline) (7), and an aminomethyl, lipophilic side group at C9 (6, 8). These specific side groups of omadacycline enhance the drug's antimicrobial activity compared with first-generation tetracycline (6), prevent recognition of omadacycline by many bacterial efflux pumps (modification at C7), and render it insensitive to alterations of the 30S ribosomal subunit of the bacterial ribosome (C9) (9).

Since efflux pumps and ribosomal protection are the most commonly acquired tetracycline resistance mechanisms, omadacycline is considered to be a good candidate for the treatment of ABSSSI and CABP infections caused by multidrug-resistant strains (7). Although omadacycline is a very potent drug and resistance toward this drug is infrequent, recent research has shown that the presence of *tet (X)* tetracycline destructases confers high-level tigecycline resistance, and it appears to be a threat to all third-generation tetracyclines (8) in GN bacteria (10, 11).

Omadacycline is metabolically stable and does not have cross-resistance with beta-lactam antibiotics, aminoglycosides, polymyxins, and fluoroquinolones (5).

Another advantage of omadacycline is that of having 2 formulations allowing for the possibility of being administered orally or intravenously. Recent studies indicated the effectiveness of an oral-only omadacycline regimen, potentially reducing hospital admissions and associated costs (12, 13). From the series of new antimicrobials recommended to treat multi-drug resistant GN bacterial infections, omadacycline is the only one to treat both CABP and ABSSSI (14).

The objective of this multisite study was to investigate the performance of the VITEK 2 AST-GN omadacycline test using clinical isolates provided and tested by external sites. The MIC VITEK 2 card results (associated with VITEK 2 Systems) were compared to the MIC results obtained by the Clinical and Laboratory Standards Institute (CLSI) broth microdilution (BMD) reference method (15). To evaluate the performance of the omadacycline test, in addition to clinical isolates, testing of challenge and reproducibility sets, accompanied by quality control (QC) testing, were also performed following the FDA (16) and ISO (17) requirements.

## MATERIALS AND METHODS

The local (CMI, CCF, IUSM, IHMA, as listed in Table S1) Institutional Review Board (IRB) granted permission to perform the study for each study site. In every case, the sites obtained an IRB approval for exemption (waiver), as no human subjects were included in this trial. The samples tested were bacterial microorganisms from clinical human residual specimen cultures.

### Study design.

The clinical trial was a multicenter study conducted at 4 external sites (Table S1) and 1 internal site. Clinical, challenge, reproducibility, and QC isolates were tested with VITEK 2 and BMD reference methods. Three different lots of VITEK 2 AST-GN cards, including omadacycline, were used for testing/collecting data. The cards consist of 64 wells and are miniaturized versions of the 2-fold serial (doubling) dilution technique for determining MICs by the microdilution method (18, 19). In addition to a control well which only contains a microbiological culture medium, the wells contain premeasured portions of a specific antibiotic combined with the culture medium. The omadacycline MIC range for VITEK 2 cards was ≤ 0.25 μg/mL to ≥16 μg/mL, with the following concentrations in the card: 0.5, 2, 8, 16 μg/mL (equivalent standard method concentration by efficacy in μg/mL). The cards were used in conjunction with the VITEK 2 System. The preliminary software version used was equivalent to 9.04. The algorithms used for the clinical performance evaluation testing were the same as those included in the VITEK 2 Systems 9.04 SW. The results were compared to BMD panel results following the CLSI procedure (15, 20). The BMD panels prepared at bioMérieux contained omadacycline ranging in concentration from 0.03125 μg/mL to 64 μg/mL, covering the FDA *Enterobacterales* breakpoint range (21). The BMD panels were prepared with fresh medium, no more than 12 h old, at the time of panel preparation and subsequently frozen for later use (20). The isolates were considered to have susceptible (S) interpretation to omadacycline when the MIC was ≤ 4 μg/mL, intermediate (I) when the MIC = 8 μg/mL, and resistant (R) when MIC ≥ 16 μg/mL. Four testing types were performed during the trial, and every testing type was performed at 3 different sites, as shown in Table S1.

Clinical isolates acquired from cultures processed at external clinical sites were comprised of contemporary and stock clinical isolates. Contemporary clinical isolates included clinical isolates up to 6 months after isolation from the clinical specimen and, if frozen, minimally subcultured. In contrast, stock isolates were frozen isolates with no time constraints, minimally subcultured, and selected to complement the contemporary isolates.

All clinical organisms were subcultured on 2 consecutive days onto Trypticase soy agar with 5% sheep blood (TSAB; Thermo Fisher Scientific Remel Products) and incubated at 35 ± 2°C for 18 to 24 h before testing. An appropriate number of pure culture colonies were suspended in 3 mL of sterile aqueous 0.45% NaCl to obtain the standard inoculum. The same standard inoculum (0.5 to 0.63 McFarland suspension) was used to inoculate the cards and the BMD reference method. The clinical isolates were tested once, using VITEK 2 automatic dilution mode. The performances were compared with the BMD MIC results. The BMD panels were read after the panels were incubated at 35 ± 2°C in ambient air for 16 to 20 h.

The challenge set consisting of 89 strains, including well-characterized isolates of species listed in the Indications for Use (IFU), including E. cloacae and K. pneumoniae (1), was developed at bioMérieux. The complete set was tested at each of the 3 trial sites, as shown in Table S1. All challenge isolates were subcultured twice onto TSAB and incubated appropriately at 35 ± 2°C for 18 to 24 h before testing. Each challenge isolate was tested one time using the same initial 0.5 to 0.63 McFarland suspension by VITEK 2 automatic and manual dilution modes as well as VITEK 2 Compact manual dilution mode. The results were compared to those from the BMD panels to establish the challenge performance. Challenge reference results from all participating sites were used to calculate the voted standard. The voted standard was the mode of results for the 3 sites. This voted standard was compared to the challenge VITEK 2 card results from the CMI site.

The reproducibility set was selected and supplied to the sites by bioMérieux and included 10 isolates with known on-scale results. The reproducibility set was tested with the VITEK 2 AST card at 3 sites (IHMA, IUSM, and STLCA) following the FDA guidance documents, and results were compared within the sites and between sites. All strains were tested in triplicate for each dilution mode (VITEK 2 automatic and manual and VITEK 2 Compact manual dilution modes) per day for 3 days with a different inoculum prepared for each test. Each isolate had 27 results for each method. Card results were compared to the card result mode for each organism, where the mode was calculated using only on-scale results. Best-case and worst-case reproducibility results for each site and all sites combined were calculated. Results within one doubling dilution from the mode were used to calculate the best-case.

Quality control strains listed in the CLSI M100 standard (20) were tested each day of comparative testing with both the VITEK 2 AST card and the BMD reference method. Escherichia coli ATCC 25922, along with S. aureus ATCC 29213 and E. faecalis ATCC 29212 served as QC isolates and were subcultured twice onto TSAB and incubated at 35 ± 2°C for 18 to 24 h before testing. The ancillary QC organisms, S. aureus ATCC 29213 and E. faecalis ATCC 29212, were tested by BMD reference method only to perform further QC of the BMD panels. If any QC results were out of range on the BMD panel, the testing for that day was repeated by both the VITEK 2 card and the BMD reference method. Out-of-range QC results were documented.

Escherichia coli ATCC 25922 was tested by both VITEK 2 AST card and the BMD reference method as the Package Insert QC Strain.

### Data analysis.

The performance was evaluated following the FDA (16) and ISO (17) guidance documents. Omadacycline has only FDA breakpoints (21); nevertheless, the analysis was also performed using the ISO acceptance criteria (for satisfying the registration needs for countries outside of the United States) with the same FDA breakpoints. The performance of the clinical and challenge isolates collected throughout the clinical trial was evaluated based on essential agreement (EA), category agreement (CA), minor error (mE), major error (ME), and very major error (VME) rates.

Essential Agreement (EA) occurs when the result of the BMD reference method and that of the VITEK 2 test card are within 1 doubling dilution. Category agreement (CA) occurs when the interpretation of the result (susceptible, intermediate, and resistant) of the BMD reference method is the same as the interpretation of the VITEK 2 test card. Acceptable values for EA and CA are ≥ 90%. The mE rate is defined as the percent of isolates that show intermediate MIC values by the BMD reference method but are rendered susceptible or resistant by VITEK 2 methods or show resistant or susceptible MIC values by the BMD reference method but are intermediate by VITEK 2 methods. The ME rate is defined as the percent of isolates that are susceptible by the BMD reference method but are resistant by the VITEK 2 methods (acceptance criteria ≤ 3.0%).

The VME rate is defined as the percent of isolates resistant by the BMD reference method but susceptible by the VITEK 2 method. The acceptable VME rate is ≤ 2% for FDA (refer to VITEK 2 AST-Gram-Negative Eravacycline 510[k] Decision Summary K191766) and ≤ 3.0% for ISO (17).

When applying ISO criteria, discrepancy resolution testing is allowed to be performed to resolve VME and ME. The errors were resolved by testing the isolate in triplicate on the VITEK 2 card and the reference method using separate bacterial inoculum suspensions. Error resolution was based on the reference results. The mode of the reference result replaced the original reference result when determining the new error rate. The card results were recorded for informational purposes only.

Evaluable results for EA are defined as results that have on-scale MIC values for both the reference and the omadacycline test or have on-scale values for either the reference or test that are greater than or less than 1 dilution from the reference method.

A trending analysis was conducted using the combined data (clinical and challenge) for each IFU species, namely, E. cloacae and K. pneumoniae. Species for which the difference between the percentage of isolates with higher or lower MIC values was higher than 30% and for which the confidence interval was determined to be statistically significant were considered to have evidence of significant trending and will be addressed in the product labeling.

## RESULTS

### Clinical performance of VITEK 2 omadacycline.

Clinical isolates were tested at 4 sites (Table S1). A total of 769 isolates were tested. The performances of VITEK 2 using auto dilution mode with these isolates were evaluated compared to BMD reference panel results. There were no samples excluded due to insufficient growth in the control well when run on VITEK 2 cards. The 769 clinical isolates included the following: Citrobacter freundii (15), Citrobacter koseri (34), Klebsiella aerogenes (30), E. cloacae (27), E. cloacae complex (3), E. coli (300), Klebsiella oxytoca (30), K. pneumoniae (300), and Serratia marcescens (30). Certain species are intrinsically resistant, and omadacycline is not active *in vitro* against *Morganella*, Proteus, and *Providencia* species; therefore, these species were not tested.

The majority of the isolates were contemporary (475/769; 61.8%) collected at the sites over a maximum of 6 months, minimally subcultured, and supplemented with stock isolates.

The BMD MIC values showed that from the total clinical isolate pool, 95.7% (736/769) were susceptible isolates, 2.6% (20/769) were intermediate, and 1.7% (13/769) were resistant.

EA, CA, and error rates were calculated for all *Enterobacterales* and for individual species.

The performance following ISO 20776-2 criteria (17) (including all Enterobacterales tested) was calculated before (Table 1) and after error resolution (Table S2). The performance after error resolutions was 97.9% (753/769) for EA and 97.1% (747/769) for CA. There were no VME or ME recorded. The performance by species was excellent, in every case EA and CA being higher than 90%, with no VME or ME. Out of 759 evaluable results, 743 were in EA; therefore, the percentage of evaluable results in EA was 97.9%.

**TABLE 1:** VITEK 2 Omadacycline Performance by Species Before Error Resolution

Org. Source	Organism	#	# EA^*a*^	% EA	# Eval^*b*^	# (%) EA^*a*^ Eval.^*b*^	# CA^*c*^	% CA^*c*^	# S^*d*^	# I^*e*^	# R^*f*^	# (%) VME^*g*^	# (%) ME^*h*^	# (%) mE^*i*^
Clinical Automatic Dilution	C. freundii	15	15	100.0	15	15 (100)	14	93.3	14	1	0^*j*^	NA	0 (0.0)	1 (6.7)
	*C. koseri*	34	34	100.0	34	34 (100)	34	100.0	34	0	0^*j*^	NA	0 (0.0)	0 (0.0)
	E. cloacae	27	25	92.6	25	23 (92.0)	26	96.3	24	0	3	0 (0.0)	0 (0.0)	1 (3.7)
	E. cloacae complex	3	3	100.0	3	3 (100)	3	100.0	3	0	0^*j*^	NA	0 (0.0)	0 (0.0)
	E. coli	300	294	98.0	299	293 (98.0)	300	100.0	300	0	0^*j*^	NA	0 (0.0)	0 (0.0)
	K. aerogenes	30	30	100.0	30	30 (100)	30	100.0	29	1	0^*j*^	NA	0 (0.0)	0 (0.0)
	K. oxytoca	30	30	100.0	30	30 (100)	30	100.0	30	0	0^*j*^	NA	0 (0.0)	0 (0.0)
	K. pneumoniae	300	293	97.7	293	286 (97.6)	282	94.0	274	15	11	1 (9.1)	0 (0.0)	17 (5.7)
	S. marcescens	30	28	93.3	30	28 (93.3)	28	93.3	28	2	0^*j*^	NA	0 (0.0)	2 (6.7)
	Clinical Total	769	752	97.8	759	742 (97.8)	747	97.1	736	19	14	1 (7.1)	0 (0.0)	21 (2.7)
Challenge Automatic Dilution	E. cloacae	35	35	100.0	25	25 (100)	34	97.1	22	3	10	0 (0.0)	0 (0.0)	1 (2.9)
	E. cloacae complex	5	5	100.0	4	4 (100)	5	100.0	4	0	1	0 (0.0)	0 (0.0)	0 (0.0)
	K. pneumoniae ssp*. pneumoniae*	7	7	100.0	2	2 (100)	7	100.0	1	1	5	0 (0.0)	0 (0.0)	0 (0.0)
	K. pneumoniae	42	42	100.0	24	24 (100)	38	90.5	15	6	21	0 (0.0)	0 (0.0)	4 (9.5)
	Challenge Total	89	89	100.0	55	55 (100)	84	94.4	42	10	37	0 (0.0)	0 (0.0)	5 (5.6)
Combined	Overall	858	841	98.0	814	797 (97.9)	831	96.9	778	29	51	1 (2.0)	0 (0.0)	26 (3.0)

a EA (Essential Agreement).

b Eval. (Evaluable).

c CA (Category Agreement).

d S (Susceptible).

e I (Intermediate).

f R (Resistant).

g VME (Very Major Error).

h ME (Major Error).

i mE (minor Error).

j Resistant strains were not available at the time of testing.

The performance following FDA criteria using IFU species, including only K. pneumoniae and E. cloacae, was 97.3% (321/330) for EA, and 94.2% (311/330) for CA. There was 1 VME (1/14; 7.1%) and no MEs (Table 2). Out of 321 evaluable results, 312 were within EA; therefore, the percentage of evaluable results in EA was 97.2%.

**TABLE 2:** VITEK 2 Omadacycline IFU Performance by Species

Org. Source	Organism	#	# EA^*a*^	% EA^*a*^	# Eval^*b*^	# (%) EA^*a*^ Eval.^*b*^	# CA^*c*^	% CA^*c*^	# S^*d*^	# I^*e*^	# R^*f*^	#(%) VME^*g*^	#(%) ME^*h*^	#(%) mE^*i*^
Clinical Automatic Dilution	E. cloacae	27	25	92.6	25	23 (92.0)	26	96.3	24	0	3	0 (0.0)	0 (0.0)	1 (3.7)
	E. cloacae complex	3	3	100.0	3	3 (100)	3	100.0	3	0	0^*j*^	NA	0 (0.0)	0 (0.0)
	K. pneumoniae	300	293	97.7	293	286 (97.6)	282	94.0	274	15	11	1 (9.1)	0 (0.0)	17 (5.7)
	Clinical Total	330	321	97.3	321	312 (97.2)	311	94.2	301	15	14	1 (7.1)	0 (0.0)	18 (5.5)
Challenge Automatic Dilution	E. cloacae	35	35	100.0	25	25 (100)	34	97.1	22	3	10	0 (0.0)	0 (0.0)	1 (2.9)
	E. cloacae complex	5	5	100.0	4	4 (100)	5	100.0	4	0	1	0 (0.0)	0 (0.0)	0 (0.0)
	*K.pneumoniae* ssp. *pneumoniae*	7	7	100.0	2	2 (100)	7	100.0	1	1	5	0 (0.0)	0(0.0)	0 (0.0)
	K. pneumoniae	42	42	100.0	24	24 (100)	38	90.5	15	6	21	0 (0.0)	0 (0.0)	4 (9.5)
	Challenge Total	89	89	100.0	55	55 (100)	84	94.4	42	10	37	0 (0.0)	0 (0.0)	5 (5.6)
Combined	Overall	419	410	97.9	376	367(97.6)	395	94.3	343	25	51	1 (2.0)	0 (0.0)	23 (5.5)

a EA (Essential Agreement).

b Eval. (Evaluable).

c CA (Category Agreement).

d S (Susceptible).

e I (Intermediate).

f R (Resistant).

g VME (Very Major Error).

h ME (Major Error).

i mE (minor Error).

j Resistant strains were not available at the time of testing.

### Challenge performance of VITEK 2 omadacycline.

Challenge isolates were tested at 3 sites (CMI, CCF, and STLCA). A total of 89 isolates were tested and results for all 89 isolates are available. The 89 challenge stock isolates included the following E. cloacae (35), E. cloacae complex (5), K. pneumoniae (42), and K. pneumoniae ssp. *pneumoniae* (7).

The BMD MIC values showed that 47.2% (42/89) of isolates were susceptible, 11.2% (10/89) were intermediate, and 41.6% (37/89) were resistant. The performances of the challenge isolates were evaluated on VITEK 2 using auto and manual dilution modes, and on VITEK 2 Compact (manual) and were compared to BMD reference panel results.

For VITEK 2 using auto dilution mode, the EA for challenge isolates was 100% (89/89), and the CA was 94.4% (84/89). There were no ME or VME, and only 5 mE were observed. All individual species had EA and CA higher than 90% (Tables 1 and 2 and Table S2). There were 55 evaluable results, all in EA; therefore, the percentage of evaluable results in EA was 100%.

For VITEK 2 using manual dilution mode, the EA for challenge isolates was 98.9% (88/89), and the CA was 91.0% (81/89). There were no ME or VME. All individual species had EA and CA higher than 90% (data not shown).

VITEK 2 Compact manual dilution mode provided an EA for challenge isolates of 100% (89/89), and CA of 92.1% (82/89). There were no ME or VME. All individual species had EA and CA higher than 90% (data not shown).

### Overall (clinical and challenge combined) performance of VITEK 2 omadacycline.

The overall performance of clinical and challenge isolates combined was evaluated on VITEK 2 using auto dilution mode compared with BMD reference panel results. The BMD MIC values showed that 90.7% (778/858) of isolates were susceptible, 3.5% (30/858) were intermediate, and 5.8% (50/858) were resistant.

The performance following ISO criteria (18) (including all *Enterobacterales* tested) after error resolutions was 98.1% (842/858) for EA, and 96.9% (831/858) for CA. There were no VME or ME (Table S2.) Out of 814 evaluable results, 798 were in EA; therefore, the percentage of evaluable results in EA was 98.0%.

The performance by species satisfied the acceptance criteria; in every case, EA and CA were higher than 90%, with no VME or ME, as listed in Table S2.

The overall performance for FDA IFU species, including only K. pneumoniae and E. cloacae, was 97.9% (410/419) for EA and 94.3% (395/419) for CA. Of 376 evaluable results, 367 were in EA; therefore, the percentage of evaluable results within EA was 97.6%. There was 1 VME (1/51; 2.0%) and no ME (Table 2).

### Time of call.

The card incubation time was recorded for all 3 modes: VITEK 2, using auto and manual dilution modes, and on VITEK 2 Compact manual, for all 859 challenge and clinical isolates. The mean incubation time for clinical isolates was 7.34 h, with a maximum standard deviation of 1.24. The incubation time exceeded 16 h only three times (3/858; 0.3%) for 2 K. pneumoniae isolates (16.73 h; 17.07 h) and 1 C. freundii isolate (17.75 h). Statistical data for all *Enterobacterales* included in the trial are presented in Table S3.

### Reproducibility of VITEK 2 omadacycline.

To assess the precision of the test, 10 isolates with on-scale MICs (E. cloacae [1], K. pneumoniae [1], and K. pneumoniae ssp. *pneumoniae* [8]) were tested in triplicate each day for 3 days at 3 clinical trial sites (IUSM, IHMA, and STLCA) in accordance with FDA (16) and ISO requirements (17). Reproducibility was evaluated using VITEK 2 auto and manual dilution modes and VITEK 2 Compact manual dilution mode. Each isolate had 27 results for each method. For each organism, the card MIC result was compared to the card MIC result mode. The differences between the card results and the card results mode for all 270 tests are presented in Table S4.

The best-case assumes that the MIC value for off-scale organism values are within 1 doubling dilution of the mode, while the worst-case assumes that the MIC value for off-scale organisms is greater than 1 doubling dilution from the mode. The VITEK 2 omadacycline reproducibility for automatic dilution was 100%; (270/270; best-case) and 99.3% (268/270; worst-case). The VITEK 2 omadacycline reproducibility for manual dilution was 99.6% (269/270; best-case) and 99.3% (268/270; worst-case). The VITEK 2 Compact omadacycline reproducibility for manual dilution was 100% (270/270; best-case) and 99.6% (269/270; worst-case). In every case, reproducibility tests met the ≥ 95% FDA and ISO criteria.

### Quality control of VITEK 2 omadacycline.

To check the performance of the VITEK 2 AST test and the BMD reference method, QC strains recommended in the CLSI M100 standard (20) were tested each day of comparative testing with the VITEK 2 (auto and manual dilution modes), VITEK 2 Compact (manual), and the BMD reference method containing omadacycline. The testing was performed a minimum of 20 times at each site (CCF, CMI, IHMA, IUSM, and STLCA) for each organism. Escherichia coli ATCC 25922 (range 0.25 μg/mL to 2 μg/mL) QC organism and 2 Gram-positive, ancillary QC organisms were tested throughout comparative testing. The ancillary QC organisms, S. aureus ATCC 29213 (range 0.12 μg/mL to 1 μg/mL) and E. faecalis ATCC 29212 (range 0.06 μg/mL to 0.5 μg/mL), were tested by BMD reference method only to perform further QC of the BMD panels. Tables S5 and S6 show the VITEK 2 QC results. In every case, the VITEK 2 results were within the range ≥ 95% of the time and met the FDA and ISO requirements (16, 17).

### Resistance characterization of isolates tested with VITEK 2 omadacycline.

Challenge isolates were sequenced to confirm the presence or absence of efflux pump genes. Out of 89 isolates, 12 isolates expressed a tetracycline resistance mechanism (Table S7). Nine K. pneumoniae isolates harbored the *tetA* gene. Five had a resistant MIC, two were intermediate, and 2 isolates were susceptible. One K. pneumoniae isolate presented the *tetB* gene and was susceptible to omadacycline, confirming expectations of being active against *Enterobacterales* expressing the TetB efflux pump (1). The efflux gene (*tetB*) is the only one that confers resistance to tetracyclines and minocycline (6). One K. pneumoniae isolate harbored the *tetD* gene and was resistant to omadacycline. One E. cloacae isolate carried the *tetG* gene and was resistant to omadacycline. The VITEK 2 MIC results for these isolates were all in perfect agreement or at least in EA with the reference results and are presented in Table S7.

### Trending analysis.

Trending analysis was calculated for the overall IFU species and for each species using the combined data (clinical and challenge) for E. cloacae and K. pneumoniae following the FDA guidance (Table S8). The trending calculation evaluated the VITEK 2 device (auto dilution mode) MIC values that were determined to be one or more doubling dilutions lower or higher than the reference method. MIC values that were off-scale for both the reference and device were not considered in the trending analysis. Enterobacter cloacae and E. cloacae complex were consolidated into a single group, E. cloacae. Klebsiella pneumoniae and K. pneumoniae ssp. *Pneumoniae* were also consolidated into a single group, K. pneumoniae. Trending was calculated for 57 E. cloacae isolates, and the percent difference was found to be 35.09% with a 95% Confidence Interval (C.I.) of 19.29% to 48.88%. Therefore, VITEK 2 AST-GN omadacycline MIC values tended to be in exact agreement or at least 1 doubling dilution higher when testing E. cloacae compared to the BMD reference method. This trending tendency will be noted in the labeling. Trending was calculated for 319 K. pneumoniae isolates, and the percent difference was found to be 4.39% with a 95% C.I. of -1.41% to 10.17%. The majority (66.77%) of the VITEK 2 AST-GN omadacycline values tended to be in exact agreement for K. pneumoniae isolates when compared to the CLSI BMD reference method, while 14.4% of K. pneumoniae isolates were at least 1 doubling dilution lower than the reference method. The overall trend was calculated for a total of 376 IFU isolates, and the percent difference was found to be 9.04% with 95% C.I. (3.55% to 14.50%) and, therefore, not significant.

## DISCUSSION

Omadacycline is one of the newly developed third-generation tetracycline-class antibiotics, potent against *Enterobacterales*, designed to overcome the most common acquired tetracycline resistance mechanisms, overexpressed efflux pumps, and ribosomal protection proteins (8). This property made the GN omadacycline quantitative test desirable on an automated AST system.

Based on the currently described study, the VITEK 2 omadacycline test received FDA 510(k) clearance in June 2022 for MIC AST of E. cloacae and K. pneumoniae isolates from ABSSSI. The cleared performance data, consisting of clinical and challenge automatic results, showed high overall essential and category agreement (EA = 97.9% and CA = 94.3%) (Table 2) when compared with the BMD reference method. Only 1 VME (K. pneumoniae isolate) was observed, which was solved upon repeat (in triplicate) testing. The card indicated consistent results at MIC = 4 μg/mL. The original reference result had a resistance interpretation (MIC = 16 μg/mL), while all 3 repeats had an intermediate interpretation (MIC = 8 μg/mL), rendering the test a minor error. Omadacycline is considered bacteriostatic (1) (due to the reversible association of tetracyclines with ribosomes [6]), making the BMD reading more difficult and subjective. Trailing growth must be ignored when reading reference BMD MICs. The repeat results proved the variability in the reference method readings. Therefore, it is unclear what the true category interpretation is for this isolate; it was not confirmed to be a true VME.

Also, the VITEK 2 omadacycline test received FDA 510(k) clearance at the same time, in June 2022, for MIC AST of K. pneumoniae isolates from CABP. K. pneumoniae is one of the common GN pathogens causing pulmonary infections associated with high rates of mortality (22).

A significant number of isolates (349) were tested, including 37 resistant isolates, to check the accuracy of detecting resistance. The essential and category agreements were EA = 98.0% (342/349) and CA = 93.7% (327/349). VME rate was 2.7% (1/37), and no MEs were registered. The VME was caused by the same K. pneumoniae isolate (tested for both the ABSSSI and CABP indications). The difference in VME rate reflects the different number of isolates considered for CABP and ABSSSI with the same breakpoints applied. The MIC distributions for the VITEK 2 test and BMD reference method are presented in Fig. S2 and S3. The number of isolates with exact MIC agreement for the omadacycline test and BMD reference method are shown on the blue background. A clustered distribution around the exact agreement can be observed, which leads to high EA values.

In addition to IFU species, other *Enterobacterales* species were tested except for *Morganella*, Proteus, and *Providencia* species which are designated as intrinsically resistant. VITEK 2 and BMD MIC results correlated well with each other and were in high EA and CA (≥ 90%), with no VMEs or Mes, for the following species: C. freundii, *C. koseri*, E. coli, K. aerogenes, K. oxytoca, and S. marcescens. After error resolution, the overall combined clinical and challenge performance was EA = 98.1% and CA = 96.9%. No ME or VME were registered. A breakdown by species and the MIC correlation are presented in Fig. S1 and Table S2 and show good agreement between VITEK 2 and BMD reference MIC values. The number of evaluable, on-scale results was high (94.9%, 814/858). EA rate of evaluable was 98.0% (798/814) and provided a clear description of the device’s accuracy and capacity to give a correct MIC value of the omadacycline test.

Moreover, the VITEK 2 test met the ISO and FDA criteria of ≥ 95% reproducibility as follows: best-case reproducibility for automatic dilution was 100%, for manual dilution was 99.6%, for Compact manual dilution was 100%, proving the high precision of the test (Table S4).

All throughout the study, the QC results were within acceptable ranges (≥ 95%) for all QC organisms.

One of the advantages of the VITEK 2 automated susceptibility method is the rapid time to result compared to the BMD method incubation time. All challenge tests were completed well in advance of 16 h (max. 11.35 h). The mean time of call for challenge isolates was the shortest for automatic mode: 7.28 h, slightly higher for VITEK 2 manual mode: 7.38 h, and Compact at 7.57 h. The incubation time for clinical isolates was below 16 h most of the time (99.6%; 766/769) and exceeded it only three times (0.4%, 3/769). The mean time of call was 7.34 h, much shorter than typical BMD overnight results. These results demonstrate that the omadacycline VITEK 2 rapid test leads to faster AST results than the BMD method, consequently improving diagnostic procedures.

A significant number of resistant isolates was tested (51), proving that VITEK 2 AST-GN omadacycline can accurately detect resistance. However, no resistant strains were tested for non-IFU species, C. freundii, *C. koseri*, E. coli, K. aerogenes, K. oxytoca, and S. marcescens, constituting a limitation of the study.

If interpretive criteria and breakpoints will be established by other committees (e.g., CLSI or EUCAST), the VITEK 2 omadacycline test will be assessed to determine performance with the new breakpoints.

The present study validated the newly developed AST-GN omadacycline test as a new dependable automated AST quantitative test, through performance, quality, and reproducibility results, which was the study’s purpose.

However, there were insufficient data to assess the level of omadacycline susceptibility when *tet* genes are present. Further studies may include isolates that harbor tetracycline resistance genes, including the *tet(X)* gene. *Tet (X)* is a gene encoding tetracycline destructases that confers high-level tigecycline resistance, and it appears to be a threat to third-generation tetracyclines (8) in GN bacteria (10, 11).

In conclusion, the results of the present multicenter clinical trial demonstrated a good correlation between VITEK 2 AST-GN omadacycline for *Enterobacterales* and the BMD reference method. Our results enable the use of the omadacycline susceptibility test in conjunction with VITEK 2 and VITEK 2 Compact Systems (SW version 9.04 and higher) as a new IVD tool to determine MICs in *Enterobacterales*. The responsible use of the new antibiotic, omadacycline, is highly encouraged to withhold and possibly avoid the development of new antimicrobial resistance patterns and to help antimicrobial stewardship programs (1, 22).
