# Supplementary material for: Multicenter Clinical Performance Evaluation of Omadacycline Susceptibility Testing of Enterobacterales on VITEK 2 Systems
Source: J Clin Microbiol. 2023 May 10;61(6):e00174-23. doi: 10.1128/jcm.00174-23 (PMC10281136; doi:10.1128/jcm.00174-23)
Supplement: Supplemental file 1 — Supplemental material. Download jcm.00174-23-s0001.docx, DOCX file, 0.09 MB [file jcm.00174-23-s0001.docx]

Supplementary Materials **(Multicenter Clinical Performance Evaluation of Omadacycline Susceptibility Testing of Enterobacterales on VITEK^®^ 2 Systems)**

## Figures

Figure S1. Frequency table – Enterobacterales - Correlation of omadacycline MIC values between VITEK 2 and BMD reference methods

Omadacycline FDA breakpoints: ≤ 4 (S), 8 (I), ≥ 16 (R). Clinical and challenge isolates (858) were tested for VITEK 2 AST-GN omadacycline and the BMD reference method*.* The number of isolates with exact MIC agreement for the omadacycline test and BMD reference method are shown on a blue background. The number of isolates constituting mEs are on a green background.

|  | **Reference** | | | | | | | | | | | | |
| --- | --- | --- | --- | --- | --- | --- | --- | --- | --- | --- | --- | --- | --- |
| **Test results** | **≤0.031** | **0.062** | **0.125** | | **0.25** | **0.5** | **1** | **2** | **4** | **8** | **16** | **32** | **≥64** |
| **≤0.25** |  |  | |  |  | 1 | 1 | 1 |  |  |  |  |  |
| **0.5** |  |  | |  |  | 15 | 57 | 1 |  |  |  |  |  |
| **1** |  |  | |  |  | 16 | 189 | 122 | 6 |  |  |  |  |
| **2** |  |  | |  |  |  | 31 | 179 | 17 |  |  |  |  |
| **4** |  |  | |  |  |  | 2 | 59 | 75 | 10 |  |  |  |
| **8** |  |  | |  |  |  |  | 1 | 5 | 18 | 5 | 1 | 3 |
| **≥16** |  |  | |  |  |  |  |  |  | 2 | 22 | 11 | 8 |
| **Evaluable results** |  |  | |  |  | 31 | 280 | 363 | 103 | 28 | 5 | 1 | 3 |

## Figure S2. Frequency table - Enterobacterales (IFU: *K. pneumoniae, E. cloacae) -* Correlation of omadacycline MIC values between VITEK 2 and BMD reference methods

Omadacycline FDA breakpoints: ≤ 4 (S), 8 (I), ≥ 16 (R). Clinical and challenge isolates (419) were tested for VITEK 2 AST-GN omadacycline and the BMD reference method*.* The number of isolates with exact MIC agreement for the omadacycline test and BMD reference method are shown on a blue background. The number of isolates constituting VME is shown on an red background, while the mEs are on a green background.

|  | **Reference** | | | | | | | | | | | | | |
| --- | --- | --- | --- | --- | --- | --- | --- | --- | --- | --- | --- | --- | --- | --- |
| **Test results** | **≤0.0312** | **0.0625** | **0.125** | | **0.25** | **0.5** | **1** | **2** | **4** | | **8** | **16** | **32** | **≥64** |
| **≤0.25** |  |  | |  |  |  |  | 1 | |  |  |  |  |  |
| **0.5** |  |  | |  |  |  |  |  |  | |  |  |  |  |
| **1** |  |  | |  |  | 1 | 32 | 30 |  | |  |  |  |  |
| **2** |  |  | |  |  |  | 27 | 142 | 4 | |  |  |  |  |
| **4** |  |  | |  |  |  | 2 | 49 | 49 | | 6 | 1 |  |  |
| **8** |  |  | |  |  |  |  | 1 | 5 | | 17 | 5 | 1 | 3 |
| **≥16** |  |  | |  |  |  |  |  |  | | 2 | 22 | 11 | 8 |
| **Evaluable results** |  |  | |  |  | 1 | 61 | 223 | 58 | | 23 | 6 | 1 | 3 |

## Figure S3. Frequency table - *K. pneumoniae -* Correlation of omadacycline MIC values between VITEK 2 and BMD reference methods

Omadacycline FDA breakpoints: ≤ 4 (S), 8 (I), ≥ 16 (R). Clinical and challenge isolates (349) were tested for VITEK 2 AST-GN omadacycline and the BMD reference method*.* The number of isolates with exact MIC agreement for the omadacycline test and BMD reference method are shown on a blue background. The number of isolates constituting VME is shown an red background, while the mEs are on a green background.

|  | **Reference** | | | | | | | | | | | | | | |
| --- | --- | --- | --- | --- | --- | --- | --- | --- | --- | --- | --- | --- | --- | --- | --- |
| **Test results** | **≤0.031** | **0.062** | **0.125** | | **0.25** | **0.5** | **1** | **2** | **4** | | | **8** | **16** | **32** | **≥64** |
| **≤0.25** |  |  | |  |  |  |  | 1 | | |  |  |  |  |  |
| **0.5** |  |  | |  |  |  |  |  |  | | |  |  |  |  |
| **1** |  |  | |  |  | 1 | 31 | 26 |  | | |  |  |  |  |
| **2** |  |  | |  |  |  | 27 | 132 | | 4 | |  |  |  |  |
| **4** |  |  | |  |  |  | 1 | 26 | 36 | | | 6 | 1 |  |  |
| **8** |  |  | |  |  |  |  | 1 | 5 | | | 14 | 5 | 1 | 2 |
| **≥16** |  |  | |  |  |  |  |  |  | | | 2 | 12 | 10 | 6 |
| **Evaluable results** |  |  | |  |  | 1 | 59 | 186 | | 44 | | 20 | 6 | 1 | 2 |

# Tables

## Table S1. Clinical Trial Sites and Associated Testing Types

| Clinical Trial Site | Testing Type |
| --- | --- |
| Clinical Microbiology Institute (CMI) | Clinical, Challenge, QC |
| Cleveland Clinic (CCF) | Clinical, Challenge, QC |
| Indiana University School of Medicine (IUSM) | Clinical, Reproducibility, QC |
| International Health Management Associates (IHMA) | Clinical, Reproducibility, QC |
| bioMérieux, Inc. (STLCA) | Challenge, Reproducibility, QC |

## Table S2. VITEK 2 Omadacycline Performance by Species After Error Resolution

| Org. Source | Organism | # | #  EA | %  EA | #  Eval. | # (%)  EA Eval. | #  CA | %  CA | #  S | #  I | #  R | #(%)  VME | #(%)  ME | #(%)  mE |
| --- | --- | --- | --- | --- | --- | --- | --- | --- | --- | --- | --- | --- | --- | --- |
| Clinical Automatic Dilution | *C. freundii* | 15 | 15 | 100.0 | 15 | 15(100) | 14 | 93.3 | 14 | 1 | 0^1^ | NA | 0(0.0) | 1(6.7) |
|  | *C. koseri* | 34 | 34 | 100.0 | 34 | 34(100) | 34 | 100.0 | 34 | 0 | 0^1^ | NA | 0(0.0) | 0(0.0) |
|  | *E. cloacae* | 27 | 25 | 92.6 | 25 | 23(92.0) | 26 | 96.3 | 24 | 0 | 3 | 0(0.0) | 0(0.0) | 1(3.7) |
|  | *E. cloacae* complex | 3 | 3 | 100.0 | 3 | 3(100) | 3 | 100.0 | 3 | 0 | 0^1^ | NA | 0(0.0) | 0(0.0) |
|  | *E. coli* | 300 | 294 | 98.0 | 299 | 293(98.0) | 300 | 100.0 | 300 | 0 | 0^1^ | NA | 0(0.0) | 0(0.0) |
|  | *K. aerogenes* | 30 | 30 | 100.0 | 30 | 30(100) | 30 | 100.0 | 29 | 1 | 0^1^ | NA | 0(0.0) | 0(0.0) |
|  | *K. oxytoca* | 30 | 30 | 100.0 | 30 | 30(100) | 30 | 100.0 | 30 | 0 | 0^1^ | NA | 0(0.0) | 0(0.0) |
|  | *K. pneumoniae* | 300 | 294 | 98.0 | 293 | 287(98.0) | 282 | 94.0 | 274 | 16 | 10 | 0(0.0) | 0(0.0) | 18(6.0) |
|  | *S. marcescens* | 30 | 28 | 93.3 | 30 | 28(93.3) | 28 | 93.3 | 28 | 2 | 0^1^ | NA | 0(0.0) | 2(6.7) |
|  | Clinical Total | 769 | 753 | 97.9 | 759 | 743(97.9) | 747 | 97.1 | 736 | 20 | 13 | 0(0.0) | 0(0.0) | 22(2.9) |
| Challenge Automatic Dilution | *E. cloacae* | 35 | 35 | 100.0 | 25 | 25(100) | 34 | 97.1 | 22 | 3 | 10 | 0(0.0) | 0(0.0) | 1(2.9) |
|  | *E. cloacae* complex | 5 | 5 | 100.0 | 4 | 4(100) | 5 | 100.0 | 4 | 0 | 1 | 0(0.0) | 0(0.0) | 0(0.0) |
|  | *K. pneumoniae* ssp*. pneumoniae* | 7 | 7 | 100.0 | 2 | 2(100) | 7 | 100.0 | 1 | 1 | 5 | 0(0.0) | 0(0.0) | 0(0.0) |
|  | *K. pneumoniae* | 42 | 42 | 100.0 | 24 | 24(100) | 38 | 90.5 | 15 | 6 | 21 | 0(0.0) | 0(0.0) | 4(9.5) |
|  | Challenge Total | 89 | 89 | 100.0 | 55 | 55(100) | 84 | 94.4 | 42 | 10 | 37 | 0(0.0) | 0(0.0) | 5(5.6) |
| Combined | Overall | 858 | 842 | 98.1 | 814 | 798(98.0) | 831 | 96.9 | 778 | 30 | 50 | 0(0.0) | 0(0.0) | 27(3.1) |

EA (Essential Agreement); Eval. (Evaluable); CA (Category Agreement); S (Susceptible); I (Intermediate); R (Resistant); VME (Very Major Error); ME (Major Error); mE (minor Error)

^1^Resistant strains were not available at the time of testing

## Table S3. Time of Call

| Sample Type | Dilution | Total Samples | Mean | Std. Dev. | Min | Max | Number of Calls > 16 Hours | Percent of Calls >16 Hours |
| --- | --- | --- | --- | --- | --- | --- | --- | --- |
| Challenge | Automatic | 89 | 7.28 | 1.04 | 5.30 | 11.35 | 0 | 0 |
| Challenge | Manual | 89 | 7.38 | 1.07 | 5.52 | 11.67 | 0 | 0 |
| Challenge | Compact | 89 | 7.56 | 1.02 | 5.78 | 10.90 | 0 | 0 |
| Clinical | Automatic | 769 | 7.34 | 1.24 | 5.55 | 17.75 | 3 | 0.4 |

## Table S4. Reproducibility Performance of VITEK 2 AST Omadacycline

| **Organism** | **Method** | **Dillution Diffrences btv. Card Result and Card Result Mode** | | | | | | | **Card Result Mode (µg/mL)** |
| --- | --- | --- | --- | --- | --- | --- | --- | --- | --- |
|  |  | **Off-scale** | **-2** | **-1** | **0** | **+1** | **+2** | **Off-scale** |  |
| *Enterobacter cloacae* | VT2 Auto-Dilution |  |  |  | 27 |  |  |  | 4 |
|  | VT2 Manual Dilution |  |  |  | 27 |  |  |  | 4 |
|  | Compact Manual Dilution |  |  |  | 27 |  |  |  | 4 |
| *Klebsiella pneumoniae* ssp*. pneumoniae* | VT2 Auto-Dilution |  |  |  | 27 |  |  |  | 4 |
|  | VT2 Manual Dilution |  |  |  | 27 |  |  |  | 4 |
|  | Compact Manual Dilution |  |  |  | 27 |  |  |  | 4 |
| *Klebsiella pneumoniae* ssp. *pneumoniae* | VT2 Auto-Dilution |  |  |  | 27 |  |  |  | 2 |
|  | VT2 Manual Dilution |  |  | 3 | 24 |  |  |  | 2 |
|  | Compact Manual Dilution |  |  |  | 27 |  |  |  | 2 |
| *Klebsiella pneumoniae* ssp*. pneumoniae* | VT2 Auto-Dilution |  |  |  | 27 |  |  |  | 8 |
|  | VT2 Manual Dilution |  |  |  | 27 |  |  |  | 8 |
|  | Compact Manual Dilution |  |  |  | 27 |  |  |  | 8 |
| *Klebsiella pneumoniae* ssp. *pneumoniae* | VT2 Auto-Dilution |  |  |  | 27 |  |  |  | 8 |
|  | VT2 Manual Dilution |  |  |  | 27 |  |  |  | 8 |
|  | Compact Manual Dilution |  |  |  | 27 |  |  |  | 8 |
| *Klebsiella pneumoniae* ssp*. pneumoniae* | VT2 Auto-Dilution |  |  |  | 27 |  |  |  | 8 |
|  | VT2 Manual Dilution |  |  |  | 27 |  |  |  | 8 |
|  | Compact Manual Dilution |  |  |  | 27 |  |  |  | 8 |
| *Klebsiella pneumoniae* ssp*. pneumoniae* | VT2 Auto-Dilution |  |  |  | 27 |  |  |  | 8 |
|  | VT2 Manual Dilution |  |  |  | 27 |  |  |  | 8 |
|  | Compact Manual Dilution |  |  |  | 27 |  |  |  | 8 |
| *Klebsiella pneumoniae* ssp*. pneumoniae* | VT2 Auto-Dilution |  |  |  | 25 |  |  | 2 | 8 |
|  | VT2 Manual Dilution |  |  |  | 26 |  |  | 1 | 8 |
|  | Compact Manual Dilution |  |  |  | 26 |  |  | 1 | 8 |
| *Klebsiella pneumoniae* ssp*. pneumoniae* | VT2 Auto-Dilution |  |  |  | 14 | 13 |  |  | 4 |
|  | VT2 Manual Dilution |  | 1 |  | 15 | 11 |  |  | 4 |
|  | Compact Manual Dilution |  |  |  | 18 | 9 |  |  | 4 |
| *Klebsiella pneumoniae* | VT2 Auto-Dilution |  |  |  | 26 | 1 |  |  | 4 |
|  | VT2 Manual Dilution |  |  |  | 26 | 1 |  |  | 4 |
|  | Compact Manual Dilution |  |  |  | 20 | 7 |  |  | 4 |
| Total | VT2 Auto-Dilution | 0 | 0 | 0 | 254 | 14 | 0 | 2 |  |
|  | Best-Case Combined Reproducibility 270/270 =100% | | | | | | | | |
| Total | VT2 Manual Dilution | 0 | 1 | 3 | 253 | 12 | 0 | 1 |  |
|  | Best-Case Combined Reproducibility 269/270 =99.63% | | | | | | | | |
| Total | Compact Manual Dilution | 0 | 0 | 0 | 253 | 16 | 0 | 1 |  |
|  | Best-Case Combined Reproducibility 270/270 =100% | | | | | | | | |

## Table S5. VITEK 2 System Quality Control Summary

| QC Strain | CLSI QC Range µg/mL | VITEK2 Card QC Range µg/mL | No (%) VITEK 2 Results Within Range | | | % Reference Results Within Range |
| --- | --- | --- | --- | --- | --- | --- |
|  |  |  | Auto | Manual | Compact |  |
| *E. coli* ATCC 25922 | 0.25– 2 | ≤0.25– 2 | 204/204  (100) | 97/98  (99.0) | 100/100  (100) | 99.5 |

## Table S6. Ancillary Quality Control Summary:

| QC Strain | CLSI  QC Range  µg/mL | Total Tested | Number Reference Results Within Range | Percent Reference Results Within Range |
| --- | --- | --- | --- | --- |
| *S. aureus* ATCC 29213 | 0.12-1 | 207 | 207 | 100% |
| *E. faecalis* ATCC 29212 | 0.06- 0.5 | 206 | 206 | 100% |

## Table S7. Strains Containing Efflux Genes

| Strain Number | Isolate Name | VITEK 2 Omadacycline MIC | Ref.  MIC | Efflux  Genes |  |
| --- | --- | --- | --- | --- | --- |
|  |  |  |  |  |  |
| 116348 | *Enterobacter cloacae* | ≥16 | 16 | tet(G) |  |
| 116350 | *Klebsiella pneumoniae* | ≥16 | 32 | tet(A) |  |
| 116356 | *Klebsiella pneumoniae* | ≥16 | 32 | tet(A) |  |
| 116359 | *Klebsiella pneumoniae* | ≥16 | 32 | tet(A) |  |
| 116385 | *Klebsiella pneumoniae* | 8 | 8 | tet(A) |  |
| 116422 | *Klebsiella pneumoniae* | 8 | 16 | tet(A) |  |
| 116424 | *Klebsiella pneumoniae* | 4 | 4 | tet(A) |  |
| 116430 | *Klebsiella pneumoniae* | 8 | 8 | tet(A) |  |
| 116440 | *Klebsiella pneumoniae* | 4 | 4 | tet(B) |  |
| 116450 | *Klebsiella pneumoniae* | ≥16 | 16 | tet(A) |  |
| 116472 | *Klebsiella pneumoniae* | 8 | 16 | tet(D) |  |
| 118125 | *Klebsiella pneumoniae* | 4 | 4 | tet(A) |  |

## Table S8. Omadacycline Trending Analysis: VITEK 2 Auto-Dilution (Challenge and Clinical Isolates)

| Organism | Total Evaluable for Trending | ≥1 dil. | Exact | ≥1 dil. | Percent Difference (95% CI) | Trending Noted |
| --- | --- | --- | --- | --- | --- | --- |
|  |  | Lower |  | Higher |  |  |
|  |  | # (%) | # (%) | # (%) |  |  |
| *E.cloacae* | 57 | 5 | 27 | 25 | 35.09% | Yes |
|  |  | 8.77% | 47.37% | 43.86% | (19.29, 48.88) |  |
| *K. pneumoniae* | 319 | 46 | 213 | 60 | 4.39% | No |
|  |  | 14.42% | 66.77% | 18.81% | (-1.41,10.17) |  |
| Enterobacterales | 376 | 51 | 240 | 85 | 9.04% | No |
|  |  | 13.56% | 63.83% | 22.61% | (3.55, 14.50) |  |
